# Barriers to help-seeking, accessing and providing mental health support for medical students: a mixed methods study using the candidacy framework

**DOI:** 10.1186/s12913-024-11204-8

**Published:** 2024-06-15

**Authors:** Elena Sheldon, Naseeb Ezaydi, Lauren Desoysa, Jasmine Young, Melanie Simmonds-Buckley, Prof Daniel Hind, Prof Chris Burton

**Affiliations:** 1 Division of Population Health, School of Medicine and Population Health, The University of Sheffield, Sheffield, UK; 2 School of Medicine and Population Health, The University of Sheffield, Sheffield, UK; 3 Department of Psychology, The University of Sheffield, Sheffield, UK; 4 Clinical Trials Research Unit, Sheffield Health and Related Research, University of Sheffield, Sheffield, UK

**Keywords:** Medical student, Mental health, Candidacy Framework, Access to health care, Mixed Methods Research

## Abstract

**Background:**

The mental health of medical students is a national and international problem increasing in both demand and acuity. Medical students face barriers to accessing mental health support that is clinically effective, timely and appropriate for their needs. This mixed methods study aimed to explore experiences of these barriers and the challenges to health service delivery aligned to the Candidacy Framework.

**Methods:**

One hundred three medical students studying at The University of Sheffield completed an online survey comprising the CCAPS-34 and follow-up questions about service access and use. Semi-structured interviews with a nested sample of 20 medical students and 10 healthcare professionals explored barriers to service access and provision. A stakeholder panel of medical students and professionals met quarterly to co-produce research materials, interpret research data and identify touchpoints by pinpointing specific areas and moments of interaction between a medical student as a service user and a mental health service.

**Results:**

Medical students who experienced barriers to help-seeking and accessing support scored significantly higher for psychological symptoms on the CCAPS-34. Uncertainty and fear of fitness to practice processes were important barriers present across all seven stages of candidacy. The fragmented structure of local services, along with individual factors such as perceived stigma and confidentiality concerns, limited the progression of medical students through the Candidacy Framework (a framework for understanding the different stages of a person’s journey to healthcare).

**Conclusion:**

This study outlines important areas of consideration for mental health service provision and policy development to improve access to and the quality of care for medical students.

**Supplementary Information:**

The online version contains supplementary material available at 10.1186/s12913-024-11204-8.

## Background

The mental health of medical students is a national and international problem [1], requiring urgent attention [2]. Mental health problems can emerge as early as the first year with symptoms of depression, anxiety, burnout and suicidal ideation [3, 4]. A meta-analysis of 183 studies across 43 countries showed that the prevalence of depression among medical students was 27%, with 11% of those students reporting suicidal ideation and more than 80% feeling under-supported [1]. Episodes of poor mental health are associated with adverse outcomes such as alcohol and substance abuse, self-harm and dropping out of medical school [3, 5].

Medical students face particular sets of barriers to help-seeking and accessing mental health support; less than a quarter of those with clinical levels of depression report using counselling services [6]. Barriers include stigma, perceiving a mental health problem as a weakness and beliefs about “fitness to practice” (FTP) proceedings, with presumed implications for career progression [7] and the possibility of expulsion [8]. Jadzinski et al. [9] reported a lack of understanding of what FTP expectations are for medical students and inconsistencies with Higher Education Institution (HEI) processes in managing FTP concerns. Internationally, the barriers to help-seeking, which affect medical students disproportionately, are complex and multi-faceted [10, 11].

HEIs have seen a growing demand for services to meet the mental health needs of medical students [12–14]. University support services are required to provide brief in-house support to students, including counselling or mental health centres, disability support, and wellbeing services. Longer-term or specialist support for acute mental health problems are provided by external services. The Student Services Partnership Evaluation and Quality Standards (SPEQS), developed by Sheffield and University College London, included a toolkit addressing some of the challenges to cross-sector working from a professional perspective [15]. SPEQS provides a generic groundwork that must now be tailored to understanding how professionals can better meet the specific mental health needs of medical students and the associated challenges.

Access barriers, difficulty navigating pathways and overstretched health services mean that medical students who feel able to seek help can fall between the gaps [16]. Medical students may delay approaching services until their needs are severe or impact their studies [17], and may turn to more acute care settings to access professional support [18]. Understanding the experiences of medical students who have ‘fallen through the cracks’ and the challenges to treatment access are essential to improving the quality of services [19]. The aim of this study was to examine how barriers to accessing and navigating mental health services arise and intersect with challenges to service provision in the unique context of medical student mental health.

### Theoretical framework

We adopted the Candidacy Framework developed by Mary Dixon-Woods and colleagues [20]. Candidacy represents the idea that an individual’s access to and successful use of health services is an iterative process influenced by individual, professional, organisational, structural and resource factors. It has been used to understand healthcare experiences of vulnerable groups, including persons with MS [21] and young onset dementia [22], but has not been applied to medical students who experience mental health problems. Our study explored help-seeking behaviours, access barriers and the challenges to health service delivery aligned to the Candidacy Framework.

## Methods

This study used a mixed methods sequential design, consisting of two distinct work packages: (1) quantitative survey to describe patterns of help seeking and unmet mental health needs and (2) nested semi-structured interviews to understand more nuanced aspects of accessing and delivering support. We adhered to the Good Reporting of A Mixed Methods Study (GRAMMS) guidelines (Additional Material 1) [19, 20].

Improved systems of support can only be achieved in partnership with their intended users, participating on equal terms as stakeholders [23]. Based on the reported benefits of service user involvement in mental health service development and delivery [24], a stakeholder panel of nine medical students and five professionals met quarterly to co-produce research materials, interpret research data and identify touchpoints by pinpointing specific moments and areas of interaction between a medical student as a service user and a mental health service. These touchpoints are critical for understanding the user experience and are often targets for improving satisfaction and effectiveness. Professionals were selected for involvement in the stakeholder panel based on their organisation and role. Staff from the University of Sheffield’s Medical School (*n* = 2), NHS professionals working in community mental health settings (including low-intensity and acute care provision) (*n* = 2), and a researcher specialising in the field of student mental health (*n* = 1) were approached by e-mail. Medical students with lived experience were self-selected following an advertisement that was circulated by e-mail to all medical students at The University of Sheffield’s Medical School. The stakeholder panel therefore involved a diversity of voices to ensure meaningful input throughout that was based on both professional and lived experiences.

Work Package 1 involved a cross-sectional online survey of medical students studying at School of Medicine and Population Health, The University of Sheffield. The survey included the Counselling Centre Assessment of Psychological Symptoms (CCAPS-34) [25], a 34-item instrument with seven distinct sub-scales related to psychological symptoms and distress in university students. Items are rated on a five-point Likert scale (0 = not at all like me, 4 = extremely like me) with higher scores indicating higher severity. The survey employed multiple choice questions on participant demographics, help-seeking behaviours and service use (Additional Material 2). The survey was conducted using the Qualtrics Research Suite (Qualtrics, Provo, UT), with a one-week response window from 04/11/2022 to 11/11/2022.

An email invitation was sent to all eligible medical students aged 18 or over and studying MBChB Medicine (A100) degree or MBChB Graduate Entry Medicine (A101) at The University of Sheffield. The email included a webpage link to the Participant Information Sheet and online survey. Informed consent was completed online prior to data collection. The survey link was advertised on the student intranet news feed.

To ensure confidentiality, names were not collected except where medical students consented to contact for the interviews. Data was stored on a secure file server accessible only to the research team. Descriptive statistics and one-way ANOVAs were produced using the software R version 4.2.1 to explore differences in symptom profiles between demographics, help-seeking behaviours and service use. CCAPS-34 subscales could not be calculated where participants responded with the same value for each question in the subscale. At least 33% of questions must be answered in the subscale to calculate a valid subscale score. The overall CCPAS-34 scores and subscale scores are calculated by the mean of the available items, assuming the missing data rules hold. Details on the scoring and handling of missing data for the CCPAS-34 can be found in Additional Material 3.

Medical students responding to the survey were invited to register interest in semi-structured interviews (Work Package 2). The survey therefore provided a nested cohort from which a purposive sample of medical students were approached by email. Sampling was based on those with the highest CCAPS-34 scores, or a disclosure of previous or current use of mental health services. Medical students who disclosed mental health concerns but decided not to seek help based on their responses to the multiple-choice questions on help-seeking behaviours and service use were also approached. The stakeholder panel informed sampling based on maximum variation for demographic characteristics. Professionals were contacted for interview by email based on their organisation and role. 20 medical students and ten professionals were invited to take part by e-mail that provided a Participant Information Sheet and contact details for the research team This was considered adequate for data saturation [26] using established frameworks [27] and demonstrates integration of mixed methods at the design stage.

Interviews took place using a secure internet application with an audio consent procedure. Topic guides were co-designed with the stakeholder panel (Additional Materials 4 and 5). Potential items for the topic guide were informed by theories of (non-) help-seeking in young adults [28], covering known barriers to help-seeking and risk factors. Stakeholders selected, modified and added items for inclusion in the topic guide based on their lived experiences, values and priorities. Final drafts of the topic guides were reviewed and approved by the stakeholder panel. Encrypted digital recordings were transcribed verbatim. Two researchers analysed the transcripts and all free-text survey responses within NVivo Version 12 (QSR International), using the five stages of National Centre for Social Care ‘Framework’ analysis approach: familiarisation; identifying themes; indexing; charting; interpretation and mapping [29]. This process involved using codes as a system for marking ‘parts of the text that are of special interest’ and themes as converting ‘codes into core concepts that represent the most important aspects of the results’ [30] based on the Candidacy Framework (Table 1).

**Table 1:** Adapted description of the Candidacy Framework [25]

Stages	Description
1. Identification of candidacy	Process in which a medical student considers their mental health as requiring help which legitimises them as a candidate for a mental health service
2. Navigation of services	Knowledge of mental health services provided and appraisal of seeking help and accessing services
3. Permeability of services	The ease with which a medical student can use mental health services
4. Appearance at services	Ability to assert candidacy at mental health services, articulating concerns and ‘need’ for support
5. Adjudication by healthcare professionals	Candidacy is judged by healthcare professionals, influencing a medical student’s progression through services and access to support
6. Offers of services	Offers of care or referral made by the healthcare provider, and the judgement by the medical student as to the appropriateness of the care offered
7. Operating conditions and local production of candidacy	Includes macro level factors, such as relationship aspects between University and external healthcare providers

### Ethical considerations

This project received favourable opinion from ScHARR Research Ethics Committee (049592).

## Results

### Quantitative findings

#### Survey demographics

We received 103 survey responses (103/1500, 6.9% response rate). Table 2 shows a breakdown of participant demographic categories and responses to the follow-up questions. The majority of medical students were female (66.0%), white (69.9%) and studying in their home/birth country (93.2%). Most respondents were in their first year of study (14.6%) with fewer respondents in their fifth (10.7%) and sixth years (7.8%).

**Table 2:** Participant characteristics and responses to the follow-up survey questions

	**N** (*n* = 103)	**%**
**Fee Status**		
Missing	1	0.97
Home / Birth Country	96	93.20
International	6	5.83
**Year of study**		
Missing	1	0.97
Sixth Year (Intercalated)	8	7.77
Fifth Year	11	10.68
Fourth Year	33	32.04
Third Year	16	15.53
Second Year	19	18.45
First Year	15	14.56
**Gender**		
Missing	1	0.97
Female	68	66.02
Male	32	31.07
Non-binary	1	0.97
Prefer not to say	1	0.97
**Ethnicity**		
Missing	1	0.9
Asian or Asian British	18	17.48
Black African or Black Caribbean	4	3.88
Mixed/Multiple ethnic groups	7	6.80
Other ethnic group	1	0.97
White	72	69.9
**Follow-Up Questions**		
* Have you previously received mental health support before you started studying medicine at The University of Sheffield?*		
Yes	30	29.13
No	72	69.90
Missing	1	0.97
* Have you previously received mental health support from The University of Sheffield counselling, NHS services and/or a psychological wellbeing service whilst studying at University?*		
Yes	30	29.13
No	72	69.90
Missing	1	0.97
* Are you currently receiving support from The University of Sheffield counselling, NHS services and/or psychological wellbeing service?*		
Yes	17	16.50
No	85	82.52
Missing	1	0.97
* Have you ever had concerns about your mental health and decided not to seek help from The University of Sheffield counselling, NHS services and/or other psychological wellbeing services?*		
Yes	58	56.31
No	44	42.72
Missing	1	0.97

#### CCPAS-34 scores

Of the 103 respondents, 102 completed all CCAPS items; the remaining participant completed less than 50% so were excluded in the analysis. The mean (SD) overall score for the 102 participants was 1.28 (0.62). Medical students obtained the highest score on Social Anxiety (mean = 1.96, SD = 0.94) and the lowest score on Frustration/Anger (mean = 0.76, SD = 0.68). The following subscales could not be scored because those participants responded with the same value for each question in that subscale, so their score could not be calculated: Academic Distress (8/103), Alcohol (39/103), Depression (12/103), Eating Concerns (49/103), Frustration (36/103), Generalised Anxiety (6/103) and Social Anxiety (6/103).

#### Statistical findings

The results of the statistical analyses are found in Additional Material 6. No significant findings were found between overall CCAPS-34 scores and participant demographics or the subscale scores and demographics (*p* > 0.05). Significant responses were found between overall CCAPS-34 scores and the follow-up questions, indicating those who responded ‘yes’ to those questions scored significantly higher for psychological symptoms (*p* < 0.05).

Significant responses were found between the following CCAPS-34 subscale scores and follow-up questions: Academic Distress, Depression, Frustration, Generalised Anxiety and Social Anxiety (where three out of the four questions were significant). Floor and ceiling effects for each subscale were calculated with the unadjusted mean differences (Additional Material 7).

### Qualitative findings

**Fig. 1:**
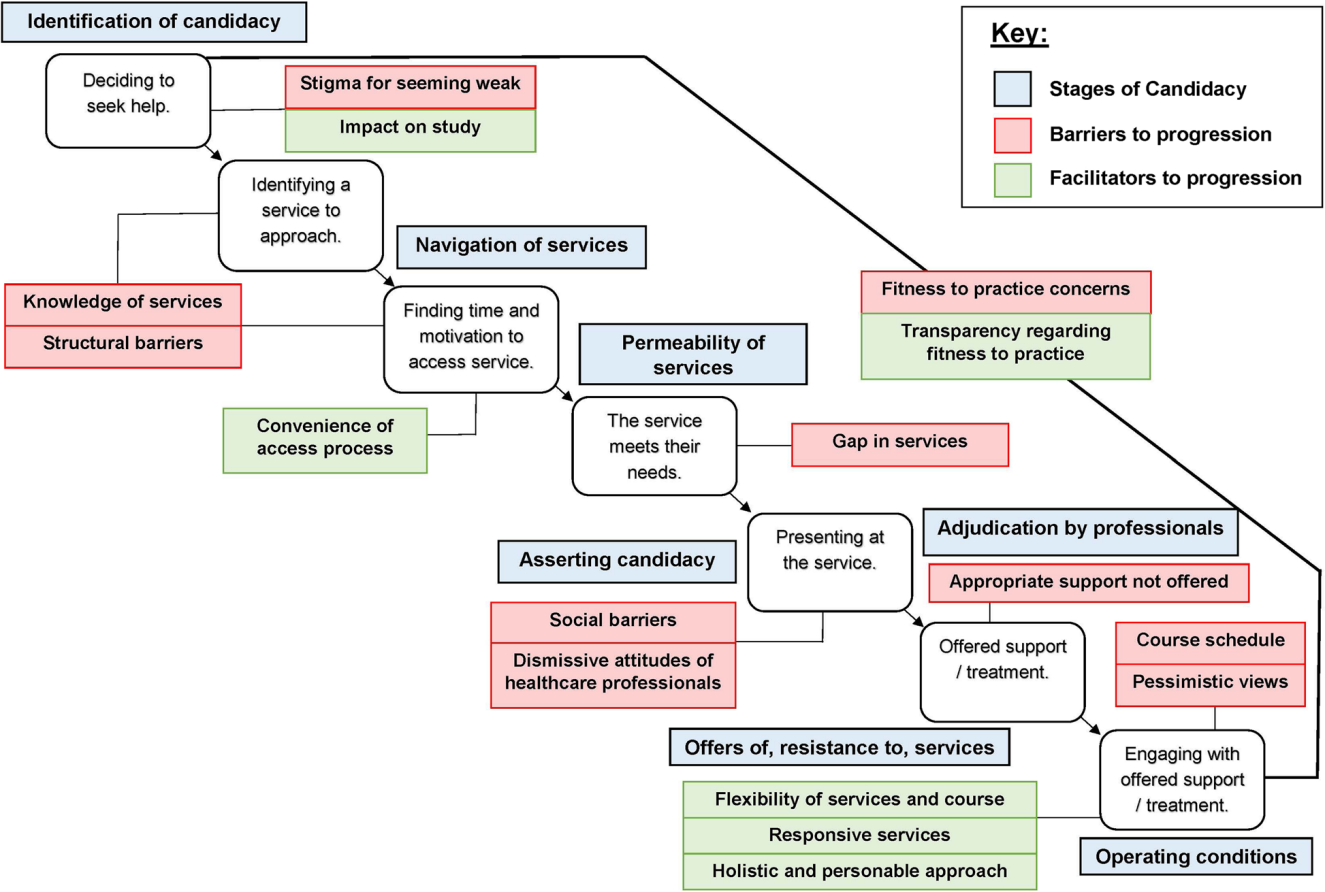
The Candidacy Framework aligned with key barriers and facilitators in the medical student pathway

Of the 103 respondents, 64 (62%) medical students consented to be contacted for interview. Interviews were conducted with 20 medical students and 10 professionals (see Table 3 for participant characteristics). As well as generic issues with access to mental healthcare for all University students, medical students face particular barriers at each stage of the Candidacy Framework (Fig. 1). Uncertainty and fear of FTP processes were mapped to all stages of candidacy as an important barrier to help-seeking and accessing support. The stigma of appearing “weak” in medical school culture; the challenges of clinical placements; and confidentiality concerns when working clinically were also highlighted as key individual-level barriers. Healthcare professionals offered insights into the fragmented structure of local services, in particular the gap in support provision between primary and secondary care.

**Table 3:** Semi-structured interview participant characteristics

*Professionals (n* = *10)*	
Profession	Mental health nurse (1) Consultant psychiatrist (2) Psychological wellbeing practitioner (1) Social worker (1) Cognitive Behavioural Therapist, University (CBT) (1) General Practitioner (1) Student affairs support staff (3)
Care setting	University counselling and therapies service (1) University health service (2) Student affairs and support (3) Sheffield Talking Therapies (1) Single Point of Access (1) Emergency department (2)
Gender	Female (10)
***Medical students (n = 20)***	
Fee status	Home / Birth country (19) International (1)
Year of study	Year 1 (4) Year 2 (2) Year 3 (4) Year 4 (6) Year 5 (4)
Ethnicity	White British (15) Asian / Asian British (2) Mixed / Multiple ethnic groups (2) Other (1)
Gender	Female (13) Male (7)
Previously used services	Sheffield Talking Therapies (3) Child and Adolescent Mental Health Service (4) Cognitive Behavioural Therapy (1) Long term private counselling (6) General Practitioner (3)

#### Fitness to practice concerns across the stages of candidacy

FTP concerns were mapped as a barrier across all Candidacy stages. Medical students who were unable to progress beyond Stage 1 reported being in denial about having a mental health problem in fear of FTP ramifications: *‘As soon as you access support you’re admitting you have a problem, so if you don’t access the support it’s just like, the denial can keep going’* (S14). Some medical students rejected referrals and offers of treatment (Stage 6): *‘I was really worried it was going to have an effect on whether or not people thought I was able to study, so I didn’t access it’* (S01).

Professionals described how FTP concerns limited data sharing opportunities where medical students chose to opt out of their information being shared with the University. In reality however, FTP proceedings were experienced as exceptional cases where symptoms were complex, acute and presented significant risks to patient safety. Crucially, professionals emphasised that concerns arise when care is *not* sought or accepted: *‘We do our best to say to students you’re not going to get into trouble for having a health problem. […]. You will get into trouble for letting it get worse and not dealing with it, because you’ve got a responsibility to be safe and practice’* (P03).

#### Stage 1: Identification of candidacy

Medical students spend a considerable amount of time in this first stage of the Candidacy Framework where they determine whether their mental health concern warrants professional support. One reason for failing to identify themselves as suitable candidates was the prioritisation of studies over mental health and wellbeing. The stigma associated with mental illness amongst peers meant that seeking help is perceived as weakness or professional risk.

*‘For people who work in the medical field or want to, then I think [the stigma] can be higher because they think it shouldn’t happen to them and they shouldn’t need support’* (P06).

#### Stage 2: Navigation of services

Once medical students identified themselves as a candidate, they determined where and how to receive appropriate care. Participants described:

- A lack of knowledge of services available for their mental health concern
- Practical barriers related to studying medicine, for example inconvenient appointment times. Often participants described that they ‘just simply don’t have the time’ (S16).

*‘That’s definitely one of the reasons why I wouldn’t access help. [...] It’s really hard to arrange appointments if you don’t know who or what you’re going to be doing’* (S18).

Professionals reflected that whilst there are a range of services accessible to medical students, they often lack awareness of what support is available. The challenge for professionals is to help medical students navigate that system by signposting to the appropriate service, arranging access via referrals or providing information on *how* to access a service.

*‘I think being able to work out which box you fit into and how to access that is more of a challenge than there not being support around.’* (P02).

#### Stage 3: Permeability of services

Most medical students reported that services were chosen based on ease of access or permeability. For those with common or ‘mild’ mental health symptoms, the University services and NHS Sheffield Talking Therapies were accessed often and easily. Medical students with acute or complex mental health problems defaulted to services that were most permeable – i.e., those with the fewest criteria to gain entry, such as the emergency department. Mental health services that would be appropriate for their level of acuity were considered inaccessible due to long waiting lists and complex referral processes.

*‘There’s a lot available for mild mental health, but for the more complex or unwell states of mental health it’s more difficult. […] It’s difficult when you fall somewhere between mild mental health and severe mental health’* (S02).

Professionals recognised the gap between care offered at a primary and secondary care level. This important challenge to service provision arose when medical students required longer-term or specialist services which have high thresholds for acceptance. Professionals reflected however, that this barrier is experienced beyond the medical student context and is recognised nationally in the UK.

*‘There’s a big gap between what’s available at the primary and secondary care level. So people with acute mental health problems where a short-term approach is not going to be helpful for, it’s hard to access psychotherapy for those people’* (P10).

#### Stage 4: Appearances at healthcare

Medical students expressed feeling uncomfortable attending appointments to discuss their mental health concerns to practitioners and described concerns that they might be known to them in an academic context.

*‘When I go to the GP, anything that I’m saying, I’m potentially saying to a future colleague that I’m potentially working with so how are they going to view me?’* (S07).

#### Stage 5: Adjudication by professionals

Medical students then faced the task of convincing healthcare providers of their candidacy for care. Many participants reported feeling invalidated or dismissed due to preconceptions held on medical students’ risk.

Healthcare provider adjudications were influenced by perceptions that there is nothing suitable to offer students and were therefore considered unfit candidates for care. In these cases, medical students reported being discharged without support or being signposted to an alternative service with lower thresholds for acceptance.

*‘They went along the lines of you’re a medical student, you’re functioning, you don’t need input from us. So they discharged me, and they discharged me without any support’* (S01).

Professionals described their frustrations however, when support cannot be offered based on a medical students’ presentation and the level of acuity required for acceptance. What was perceived as dismissive by medical students may reflect limited support options at a secondary care level.

*‘So often you want to give people something or you can identify something that could really help them but they don’t meet the criteria to access that. So that’s a big barrier’* (P01).

#### Stage 6: Offers of services

Medical students rejected offers of care due to:

- Practical barriers, such as long working hours on placement.
- Inappropriate or limited support offered that did not fit their needs.
- Support not offered within an acceptable time frame.

*‘During that time I was on placement and I was like, they’d already wriggled around my placement, I really I can’t do that again. So I declined that’* (S06).

Some medical students accepted care offers from private services to overcome these barriers. This is particularly unacceptable when considering the widening participation strategies to include medical students who are less likely to have affluent socioeconomic backgrounds and experience increased financial burden whilst studying in the UK. Professionals made efforts to overcome barriers by adapting to individual needs, for example by offering study leave so that treatment offers could be facilitated.

#### Stage 7: Operating conditions

Participants described overarching influences, including:

- Poor coordination, continuity or transference of care, especially for those who received care prior to university.
- Low capacity due to high caseloads and demand for local services.
- Limited room space; inappropriate waiting environments.

*‘I think due to waiting time, if you are at the point where you’re trying to access services and they’re just not there, it deters you from it.’* (S17).

## Discussion

### Summary of principal findings

Medical students who experienced higher levels of psychological symptoms were significantly more likely to report help-seeking concerns. This study presents key barriers to accessing mental health support at each stage of the Candidacy Framework. Uncertainty and fear of FTP processes were important barriers present across all stages. The fragmented structure of local services, along with individual factors such as stigma and confidentiality concerns, further limited the progression of medical students through the candidacy stages.

### Relationship to other research

Previous studies and policy frameworks have identified similar barriers to seeking and accessing mental health care for medical students [6–8, 26, 27], focusing primarily on individual barriers such as stigma or FTP concerns. Importantly, our findings reinforce that medical students are reluctant to disclose a mental health problem due to the feared consequences of regulatory FTP proceedings that would lead to dismissal and expulsion. The Candidacy Framework allowed us to go further by understanding how individual and service-level barriers arise and intersect with professional challenges to service provision. Applying the Framework to guide the qualitative analysis also uncovered new and unique challenges across the ‘service-user’ journey. For example, medical students with acute and complex mental health problems may fall through the gaps between primary and secondary healthcare. While there are similar studies in this field, previous findings are based on small focus groups of medical students which do not consider the perspectives of professionals working across healthcare and educational settings. To our knowledge, our study is the first to provide mixed methods findings that represent a diversity of voices and provide deeper insights into the fragmented structure of services, with care providers working across different healthcare organisations and HEIs, which are driven by different priorities. Taken together, these barriers significantly impact on candidacy and mean that medical students may feel unable to seek or access support that is clinically effective, timely and appropriate for their needs.

### Limitations and strengths

A strength of this work was that the study protocol and research materials were co-produced by a stakeholder panel of professionals and medical students with lived experience. Data was discussed by the panel to ensure views were robust, accurate and representative of values and needs. This study therefore provides an example of how working in partnership with people with lived experience and professional stakeholders can meaningfully inform our understanding of mental health service delivery and development. Another strength was the triangulation of multiple data sources to understand barriers to service access and delivery. The initial survey data uncovered how mental health symptoms may relate to help-seeking behaviours and service use. After this data was analysed, we determined how these barriers aligned with the Candidacy Framework and professionals’ experiences of service provision.

Surveying and interviewing medical students at one time point does not however, allow for an exploration of the complete student journey across a medicine degree. Potential limitations are the cross-sectional survey design, where a longitudinal approach may have allowed for a more robust view of how help-seeking may change during the academic year. We also acknowledge that the online survey was administered at the tail-end of the COVID-19 Pandemic, which may have accounted for increased psychological symptoms, such as anxiety [31]. Another limitation is the sole focus on a single UK medical school. Our low response rate may indicate a potential response bias, with medical students who have previously experienced mental health issues being more likely to participate in the survey than those who have not. We aimed for maximal variation by interviewing professionals from a range of settings and selecting medical students with different mental health profiles who had accessed a range of services. However, our findings are limited to a small sample size and reflect local context and policies – particularly in terms of how healthcare systems are configured and their operating conditions.

### Implications for healthcare services, policy-makers and further research

Asserting candidacy takes work from the service user, healthcare and University professionals and other stakeholders [32]. Our findings can help to identify groups of medical students who are at risk of ‘falling through the cracks’ in the system, which is an essential condition to prioritising resource allocation and providing accessible care. In line with guidance from MQ Mental Health Research [33], policy-makers should aim to improve the accessibility of mental health services by providing integrated high-quality care and prioritising strategies to reduce stigma. For medical schools in particular, stigma reduction strategies should provide clear FTP guidance that supports informed decision-making, personalised planning and seeking timely and appropriate support for mental health symptoms. Universities and healthcare services should further aim to address the gap between primary and secondary services by providing care that is more integrated and coordinated – particularly for medical students with complex and acute mental health problems who, based on our findings, are possibly more at risk of falling between this gap in service provision. The Sheffield Primary and Community Mental Health Transformation Programme [34] provides a local model of care aiming to inform a new way of delivering adult mental health services and break down barriers between primary and secondary care. More generally, we recommend partnership working between HEIs, healthcare services and medical students to inform service development and delivery.

Future studies should explore the experiences of specific case groups of medical students, particularly those with different types and acuity of mental health symptoms to determine how these factors influence candidacy. The MIND collaboration (10.17605/OSF.IO/48WE2) is co-producing a process map of existing service pathways to identify gaps along the student journey and is co-designing a toolkit to address some of the touchpoints and barriers identified in this research.

## Conclusions

Our findings indicate that fear of FTP processes, along with the fragmented structure of local services and individual factors such as perceived stigma, limit the progression of medical students through the Candidacy Framework. By understanding these barriers and gaps in service provision, Universities and healthcare services can be developed to better to meet medical students’ mental health needs based on their presenting problem and stage of candidacy.

### Supplementary Information

Supplementary Material 1.

Supplementary Material 2.

Supplementary Material 3.

Supplementary Material 4.

Supplementary Material 5.

Supplementary Material 6.

Supplementary Material 7.

## Data Availability

The statistical analysis plan and outputs are included as additional files. The datasets used and/or analysed during the current study are available from the corresponding author on reasonable request.

## Abbreviations

CCAPS-34: Counselling Centre Assessment of Psychological Symptoms
FTP: Fitness to practice
HEI: Higher Education Institution
NHS: National Health Service

## References

[1] Billingsley M (2015). More than 80% of medical students with mental health issues feel under-supported, says Student BMJ survey. BMJ.

[2] Coombes R (2018). Medical students need better mental health support from universities, says BMA. BMJ.

[3] Dyrbye LN, Thomas MR, Power DV, Durning S, Moutier C, Massie FS (2010). Burnout and Serious Thoughts of Dropping Out of Medical School: A Multi-Institutional Study. Acad Med.

[4] Rotenstein LS, Ramos MA, Torre M, Bradley Segal J, Peluso MJ, Guille C (2016). Prevalence of depression, depressive symptoms, and suicidal ideation among medical students a systematic review and meta-analysis. JAMA - Journal of the American Medical Association.

[5] Stallman HM (2010). Psychological distress in university students: A comparison with general population data. Aust Psychol.

[6] Givens JL, Tjia  J (2002). Depressed Medical Students’ Use of Mental Health Services and Barriers to Use. Acad Med.

[7] Chew-Graham CA, Rogers A, Yassin N (2003). “I wouldn’t want it on my CV or their records”: Medical students’ experiences of help-seeking for mental health problems. Med Educ.

[8] Winter P, Rix A, Grant A (2017). Medical Student Beliefs about Disclosure of Mental Health Issues: A Qualitative Study. J Vet Med Educ.

[9] Jadzinski M, White S, Way S, Mylod D (2023). How are fitness to practise processes applied in UK higher education institutions? − A systematic review. Nurse Educ Pract.

[10] McKerrow I, Carney PA, Caretta-Weyer H, Furnari M, Miller Juve A (2020). Trends in medical students’ stress, physical, and emotional health throughout training. Med Educ Online.

[11] Batchelor R, Pitman E, Sharpington A, Stock M, Cage E (2020). Student perspectives on mental health support and services in the UK. J Furth High Educ.

[12] Callender J, Fagin J, Jenkins G, Lester J, Smith  E (2011). Mental health of students in higher education. Acad Med.

[13] Storrie K, Ahern K, Tuckett A (2010). A systematic review: Students with mental health problems—A growing problem. Int J Nurs Pract.

[14] Taylor A (2020). Overstretched NHS services are sending suicidal students back to universities for help. BMJ.

[15] Broglia E, NK, CH, BC, SBM, KL, HG, GL, BM. Student Services Partnerships Evaluation and Quality Standards (SPEQS) toolkit. 2022. https://www.officeforstudents.org.uk/advice-and-guidance/student-wellbeing-and-protection/student-mental-health/resources/support-services/.

[16] Taylor A (2020). Overstretched NHS services are sending suicidal students back to universities for help. BMJ.

[17] Broglia E, Millings A, Barkham M (2021). Student mental health profiles and barriers to help seeking: When and why students seek help for a mental health concern. Couns Psychother Res.

[18] Hong V, Busby DR, O’Chel S, King CA (2022). University students presenting for psychiatric emergency services: Socio-demographic and clinical factors related to service utilization and suicide risk. J Am Coll Health.

[19] Tang S, Reily NM, Arena AF, Sheanoda V, Han J, Draper B (2022). Predictors of not receiving mental health services among people at risk of suicide: A systematic review. J Affect Disord.

[20] Dixon-Woods M, Cavers D, Agarwal S, Annandale E, Arthur A, Harvey J (2006). Conducting a critical interpretive synthesis of the literature on access to healthcare by vulnerable groups. BMC Med Res Methodol.

[21] Pétrin J, Finlayson M, Donnelly C, McColl MA (2021). Healthcare access experiences of persons with MS explored through the Candidacy Framework. Health Soc Care Community.

[22] Novek S, Menec VH (2021). Age, Dementia, and Diagnostic Candidacy: Examining the Diagnosis of Young Onset Dementia Using the Candidacy Framework. Qual Health Res.

[23] Skivington K, Matthews L, Simpson SA, Craig P, Baird J, Blazeby JM (2018). A new framework for developing and evaluating complex interventions: Update of Medical Research Council guidance. The BMJ.

[24] Ezaydi N, Sheldon E, Kenny A, Buck ET, Weich S (2023). Service user involvement in mental health service commissioning, development and delivery: a systematic review of service level outcomes. Health Expect.

[25] Locke BD, Buzolitz JS, Lei PW, Boswell JF, McAleavey AA, Sevig TD (2011). Development of the Counseling Center Assessment of Psychological Symptoms-62 (CCAPS-62). J Couns Psychol.

[26] Hennink M, Kaiser BN (2022). Sample sizes for saturation in qualitative research: A systematic review of empirical tests. Soc Sci Med.

[27] Malterud K, Siersma VD, Guassora AD (2016). Sample Size in Qualitative Interview Studies. Qual Health Res.

[28] Rickwood D, Deane FP, Wilson CJ, Ciarrochi J (2005). Young people’s help-seeking for mental health problems. Australian e-Journal for the Advancement of Mental Health.

[29] J R, L S. Qualitative data analysis for applied policy research. The qualitative researcher’s companion. 2002.

[30] Morgan DL (2018). Themes, Theories, and Models. Qual Health Res.

[31] Frampton NSD (2021). University mental health: life in a pandemic.

[32] Mackenzie M, Turner F, Platt S, Reid M, Wang Y, Clark J (2011). What is the “problem” that outreach work seeks to address and how might it be tackled? Seeking theory in a primary health prevention programme. BMC Health Serv Res.

[33] O’Connor RC, Worthman CM, Abanga M, Athanassopoulou N, Boyce N, Chan LF (2023). Gone Too Soon: priorities for action to prevent premature mortality associated with mental illness and mental distress. The Lancet Psychiatry.

[34] Hodgson PD, Tazzyman DA, Fryer DK. Evaluation of the Sheffield Primary and Community Mental Health Transformation Programme. 2022.

