# Supplementary material for: Barriers to help-seeking, accessing and providing mental health support for medical students: a mixed methods study using the candidacy framework
Source: BMC Health Serv Res. 2024 Jun 15;24:738. doi: 10.1186/s12913-024-11204-8 (PMC11179297; doi:10.1186/s12913-024-11204-8)
Supplement: Supplementary file 1 — Supplementary Material 1. [file 12913_2024_11204_MOESM1_ESM.docx]

**Additional File 1: Good Reporting of a Mixed Methods Study (GRAMMS)**

| **GRAMMS Reporting Guidelines** | **Line number** |
| --- | --- |
| Describe the justification for using a mixed methods approach to the research question | Lines 68-72 under Methods |
| Describe the design in terms of the purpose, priority and  sequence of methods | Lines 68-72 under Methods |
| Describe each method in terms of sampling, data collection and analysis | Lines 74-131 under Methods |
| Describe where integration has occurred, how it has occurred and who has participated in it | Lines 113-119 and 124 under Methods |
| Describe any limitation of one method associated with the present of the other method | Lines 452-455 under Limitations and Strengths |
| Describe any insights gained from mixing or integrating methods | Lines 425-431 under Discussion |
