# Supplementary material for: Barriers to help-seeking, accessing and providing mental health support for medical students: a mixed methods study using the candidacy framework
Source: BMC Health Serv Res. 2024 Jun 15;24:738. doi: 10.1186/s12913-024-11204-8 (PMC11179297; doi:10.1186/s12913-024-11204-8)
Supplement: Supplementary file 2 — Supplementary Material 2. [file 12913_2024_11204_MOESM2_ESM.docx]

**Additional File 2**: **Online survey follow up questions**

The following questions are about whether you have ever tried to access or have received support from mental health services before and/or during your time at the University of Sheffield.

1. Have you *previously* received mental health support before you started studying medicine at The University of Sheffield?

Yes / No

1. Have you *previously* received mental health support from The University of Sheffield’s counselling, NHS services* and/or a psychological wellbeing service whilst studying at University?

Yes / No

1. Are you *currently* receiving support from The University of Sheffield’s counselling, NHS services* and/or psychological wellbeing service?

Yes / No

1. Have you ever had concerns about your mental health and decided *not* to seek help from The University of Sheffield’s counselling, NHS services* and/or other psychological wellbeing services?

Yes / No
