# Supplementary material for: Barriers to help-seeking, accessing and providing mental health support for medical students: a mixed methods study using the candidacy framework
Source: BMC Health Serv Res. 2024 Jun 15;24:738. doi: 10.1186/s12913-024-11204-8 (PMC11179297; doi:10.1186/s12913-024-11204-8)

**Additional File 3: Statistical Analysis Plan (SAP)**

Contents Page:

**1 INTRODUCTION**………………………………………………………………………………….1

**2 STUDY METHODS**……………………………………………………………………………….1

**3 STATISTICAL PRINCIPLES**…………………………………………………………………….1

**4 TRIAL POPULATION**………………………………………………………………………….....3

**5 ANALYSIS**…………………………………………………………………………………………4

**6 REFERENCES**…………………………………………………………………………………….7

**7 APPENDICES**……………………………………………………………………………………..8

Abbreviations:

**CCAPS 34** Counseling Center Assessment of Psychological Symptoms

**CTRU** Clinical Trials Research Unit

**MIND** Medical Student Mental Health Study

**SAP** Statistical Analysis Plan

**SD** Standard deviation

**SOP** Standard Operating Procedure

**1 INTRODUCTION**

This SAP is written in conjunction with standard operating procedures (SOPs) from the University of Sheffield Clinical Trials Research Unit (CTUR) and study documents (Protocol). The SAP will guide the statistical analysis of all quantitative outcomes in order to answer the objectives of the study.

- 1. **BACKGROUND AND RATIONAL**

Mental health problems are prevalent among medical students, with a global prevalence of 27%. Access gaps, difficulty navigating pathways and resource constraints mean that medical students who do feel able to seek help can fall between the gaps. Understanding the experiences of medical students and the challenges to service provision is essential for overcoming barriers to access and improving the quality of existing services.

- 1. **OBJECTIVES**

This is a sequential mixed-methods study incorporating 4 work packages:

1. A cross-sectional online survey (CCAPS-34, demographic and service use questions) to characterise the population of at-risk medical students
2. A stakeholder panel of medical students and professional stakeholders to co-design research materials and re-design support systems
3. Semi-structured interviews with a nested sample of medical students and professional stakeholders
4. A co-designed process map based on multi-sector service pathways to determine where system failures may occur
5. **STUDY METHODS**

**2.1 STUDY DESIGN**

This study is a sequential mixed methods study design to characterise the mental health profiles of medical students and understand barriers to accessing mental health support. Statistical analysis will be used for the cross-sectional survey results collected in Work Package 1.

**2.2. SAMPLE SIZE**

The cross-sectional survey collected 102 responses via Qualtrics. Responses were downloaded as a CSV file and imported into IBM SPSS Statistics package 28.

**2.3. TIMING OF ANALYSIS**

The cross-sectional survey will be circulated by email to medical students on Wednesday 2^nd^ November 2022. The Qualtrics survey will remain open for 1 week. Response data will be analysed in December 2022 and presented to the stakeholder panel early 2023.

1. **STATISTICAL PRINICIPLES**

Summaries of CCAPS-34 scores will comprise the total scores and subscale scores, mean, median, standard deviation, minimum and maximum as appropriate for the distributional form of the data. Summaries of categorical variables (e.g. demographic data) will comprise the number and percentage of participants in each category.

- 1. **CONFIDENCE INTERVALS AND P-VALUES**

The 95% confidence intervals for the differences in CCAPS-34 symptom profiles between demographic characteristics and the closed-ended questions on mental health concerns, help-seeking behaviours, service access and use. Significant differences will be declared if the associated p-value is less than 0.05.

1. **TRIAL POPULATION**
   1. **ELIGIBLITY**

The following inclusion and exclusion criteria must be met in order to be considered eligible for the study.

- - 1. **INCLUSION CRITERIA**

1. Participant of any gender, aged 18 years old and over
2. Participants must be studying MBChB Medicine (A100) degree or the MBChB Graduate Entry Medicine (A101) degree at The Medical School, The University of Sheffield
   - 1. **EXCLUSION CRITERIA**
3. Participants aged below the age of 18 years
4. Participants who are not studying at The Medical School, The University of Sheffield
5. Significant language barriers, which are likely to affect the participant’s understanding of the study, or the ability to complete outcome questionnaires
6. Participants who are unable to comply with the study protocol
   1. **RECRUITMENT**

All medical students who are studying at The Medical School, The University of Sheffield will be invited by email to take part in an online questionnaire study asking about their mental health symptoms, help-seeking behaviours and service use. The email will contain an anonymous web-link to the participant information sheet, the consent form and surveys. The web-link will remain open for seven days. The email will be circulated using the University’s myAnnounce system during the first semester of academic year 2022-23. Participation is voluntary.

- 1. **DEMOGRAPHIC CHARACTERISTICS**

Demographic data will be collected and presented as categorical variables including the number and percentage of participants in each category. Demographic data will be assessed for comparability between groups and compared to CCAPS-34 profiles and service use data. The following summaries will be presented:

- Fee status (home/birth country, international);
- Year of study (1-6),
- Gender (Female, male, non-binary, rather not specify),
- Ethnicity (White, Asian or Asian British, Black African or Black Caribbean, Mixed/Multiple ethnic groups, Other ethnic group, Rather not specify)

**5 ANALYSIS**

**5.1 OUTCOME DEFINITIONS**

**5.1.1 PRIMARY OUTCOME**

The Counseling Center Assessment of Psychological Symptoms (CCAPS-34; Locke et al. 2012) is a 34-item instrument measuring psychological symptoms and distress in university student samples. See Appendix 1 for the CCAPS-34 instrument. There are seven subscales including:

1. Depression
2. Generalised anxiety
3. Social anxiety
4. Academic distress
5. Eating concerns
6. Frustration/anger
7. Alcohol use

See Appendix 2 for items sorted by subscale.

Items are rated on a 5 point Likert scale (0 = not at all like me, 4 = extremely like me) with higher scores indicating higher severity of mental distress. Some items are reverse-scored prior to computing the subscales (see Appendix 2).

To score the CCAPS-34 manually, averages of the item scores for each subscales will be calculated using the MEAN function to account for any missing item responses. This will give a subscale raw score.

**5.1.2 SECONDARY OUTCOMES**

The CCAPS-34 is followed by demographic questions (see section 4.3) and additional questions on mental health concerns, help-seeking behaviours, service access and use. Questions are as follows:

Have you *previously* received mental health support before you started studying medicine at The University of Sheffield?

Yes / No

Have you *previously* received mental health support from The University of Sheffield’s counselling, NHS services* and/or a psychological wellbeing service whilst studying at University?

Yes / No

Are you *currently* receiving support from The University of Sheffield’s counselling, NHS services* and/or psychological wellbeing service?

Yes / No

Have you ever had concerns about your mental health and decided *not* to seek help from The University of Sheffield’s counselling, NHS services* and/or other psychological wellbeing services?

Yes / No

*Examples of services may include, but are not limited to, your GP, Sheffield IAPT, Single Point of Access (SPA)

Responses will be multiple choice but where participants indicate ‘Yes’ an open-ended free text box will prompt for further elaboration of students’ concerns and reasons for seeking or avoiding help.

**5.2 ANALYSIS METHODS**

**5.2.1 PRIMARY OUTCOME**

CCAPS-34 data will be used to characterise medical student mental health profiles. Means and standard deviations on CCAPS-34 total scores and subscale scores will identify patterns of symptom severity. Ceiling effects will be shown based on the percentage of students that obtained a maximum score (4 = extremely like me) for each individual subscale. Floor effects will be shown based on the percentage of students who obtained a minimum score (0 = not at all like me) for each subscale.

**5.2.2. SECONDARY OUTCOMES**

One- and two-way ANOVAs and post hoc simple effect analyses (Bonferroni-corrected) will be used to explore differences in symptom profiles between demographic characteristics and the additional follow-up questions on mental health concerns, help-seeking behaviours, service access and use.

The first analyses will explore differences across demographic characteristics and symptoms using a one-way ANOVA. For example, is there a significant difference between years of study and CCAPS-34 scores on particular subscales (such as, medical students in Year 1 have significantly higher scores on the social anxiety subscale).

| **Independent variable (nominal data coded with values)** | **Dependent variable (ordinal data)** |
| --- | --- |
| Fee status (  Home/birth country = 1  International 2 | CCAPS-34 subscales 1-7 |
| Year of study (1-6),  Year 1 = 1  Year 2 = 2  Year 3 = 3  Year 4 =4  Year 5 = 5  Year 6 = 6 | CCAPS-34 subscales 1-7 |
| Gender  0 = Female  1 = Male  2 = Non binary  3 = Rather not specify | CCAPS-34 subscales 1-7 |
| Ethnicity  1= White  2= Asian or Asian British  3= Black African or Black Caribbean  4= Mixed/Multiple ethnic groups  5= Other ethnic group, Rather not specify) | CCAPS-34 subscales 1 -7) |

The second analyses will explore differences across help-seeking, service use and CCAPS-34 symptoms. For example, exploring whether there is a significant difference in CCAPS-34 subscale scores between those who have previously received mental health support compared to those who have not.

| **Independent variable (nominal data coded with values)** | **Dependent variable (ordinal data)** |
| --- | --- |
| Have you *previously* received mental health support before you started studying medicine at The University of Sheffield?  1 = Yes  2 = No | CCAPS-34 subscales 1-7 |
| Have you *previously* received mental health support from The University of Sheffield’s counselling, NHS services* and/or a psychological wellbeing service whilst studying at University?  1 = Yes  2 = No | CCAPS-34 subscales 1-7 |
| Are you *currently* receiving support from The University of Sheffield’s counselling, NHS services* and/or psychological wellbeing service?  1 = Yes  2 = No | CCAPS-34 subscales 1-7 |
| Have you ever had concerns about your mental health and decided *not* to seek help from The University of Sheffield’s counselling, NHS services* and/or other psychological wellbeing services?  1 = Yes  2 = No | CCAPS-34 subscales 1 -7) |

**5.3 MISSING DATA**

If more than 50% of all CCAPS-34 items are missing, or if all item values are the same (e.g. all 1s) then the subscale scores cannot be calculated. In addition, individual subscales cannot be calculated if 33% or more of the subscale items are missing.

**5.3 STATISTICAL SOFTWARE**

Quantitative analysis will be performed in IBM SPSS Statistics package 28.

**6 REFERENCES**

Locke, B. D., McAleavey, A. A., Zhao, Y., Lei, P.-W., Hayes, J. A., Castonguay, L. G., Li, H., Tate, R., & Lin, Y.-C. (2012). Development and initial validation of the Counseling Center Assessment of Psychological Symptoms-34 (CCAPS-34). *Measurement and Evaluation in Counseling and Development, 45,* 151- 169. doi:10.1177/074817561143264

**TRIAL DOCUMENTS**

| **Title** | **Version** | **Date** | **Location** |
| --- | --- | --- | --- |
| Study Protocol | 1.1 | 22^nd^ September 2022 | X:\HAR_PR\PR\MIND_Study\General\1. Study Documents\1.1 Study protocol |
| Data Management Plan | 1.1 | 22^nd^ September 2022 | X:\HAR_PR\PR\MIND_Study\General\11. Data Collection and Management\11.1 Data Collection Guidance |

**CTRU Standard Operation Procedures**

| **Title** | **Version** | **Date** | **Location** |
| --- | --- | --- | --- |
| ST001 The Statistical Analysis Plan | 6.0 | 10^th^ December 2021 | X:\ScHARR\CTRU_SOPs_Current |
| ST006 Undertaking a Statistical Analysis | 3.0 | 8^th^ Feb 2021 |  |

**7 APPENDICES**

**Appendix 1. Copy of the CCAPS-34**


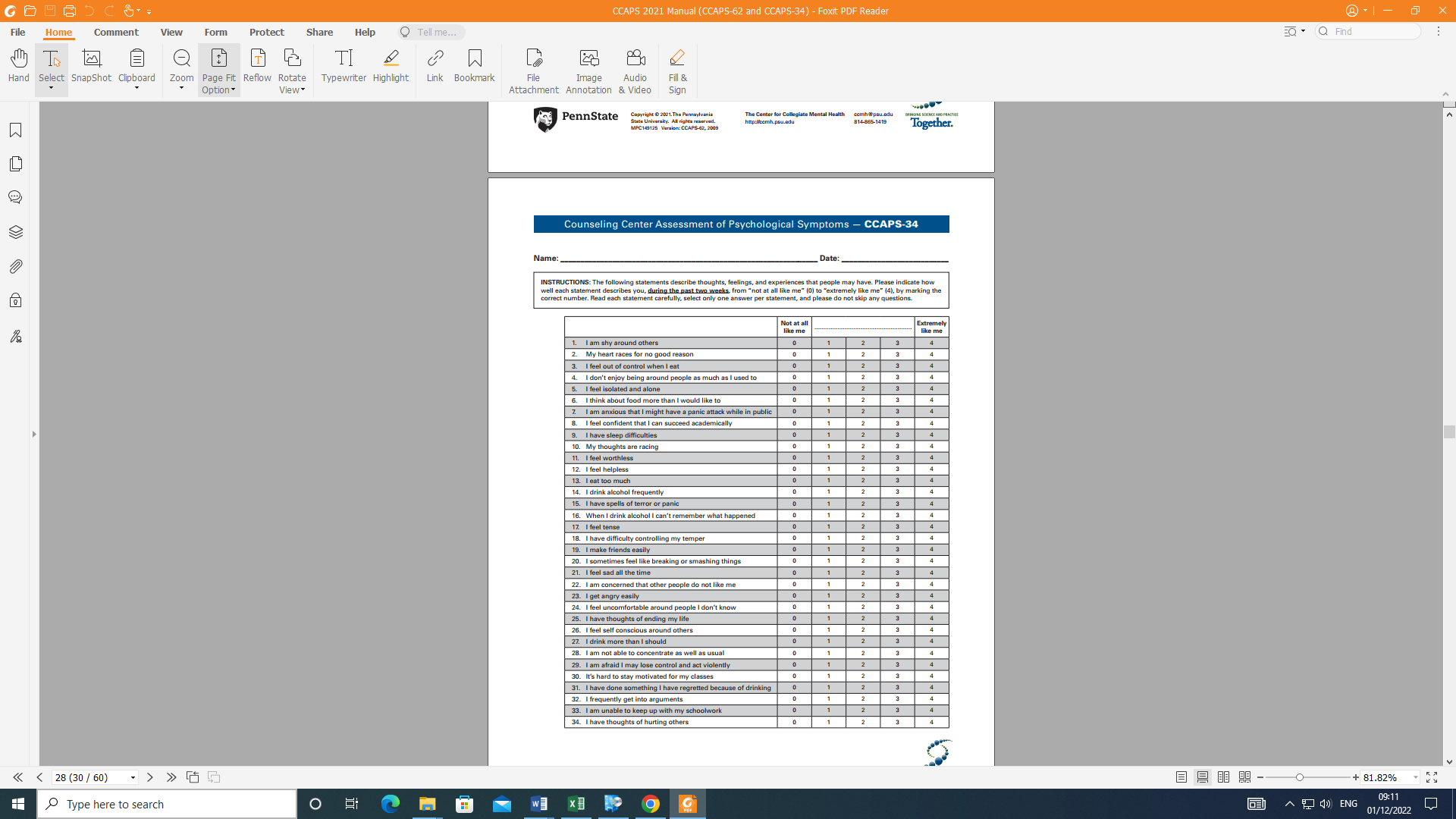


**Appendix 2. Items sorted by subscale**


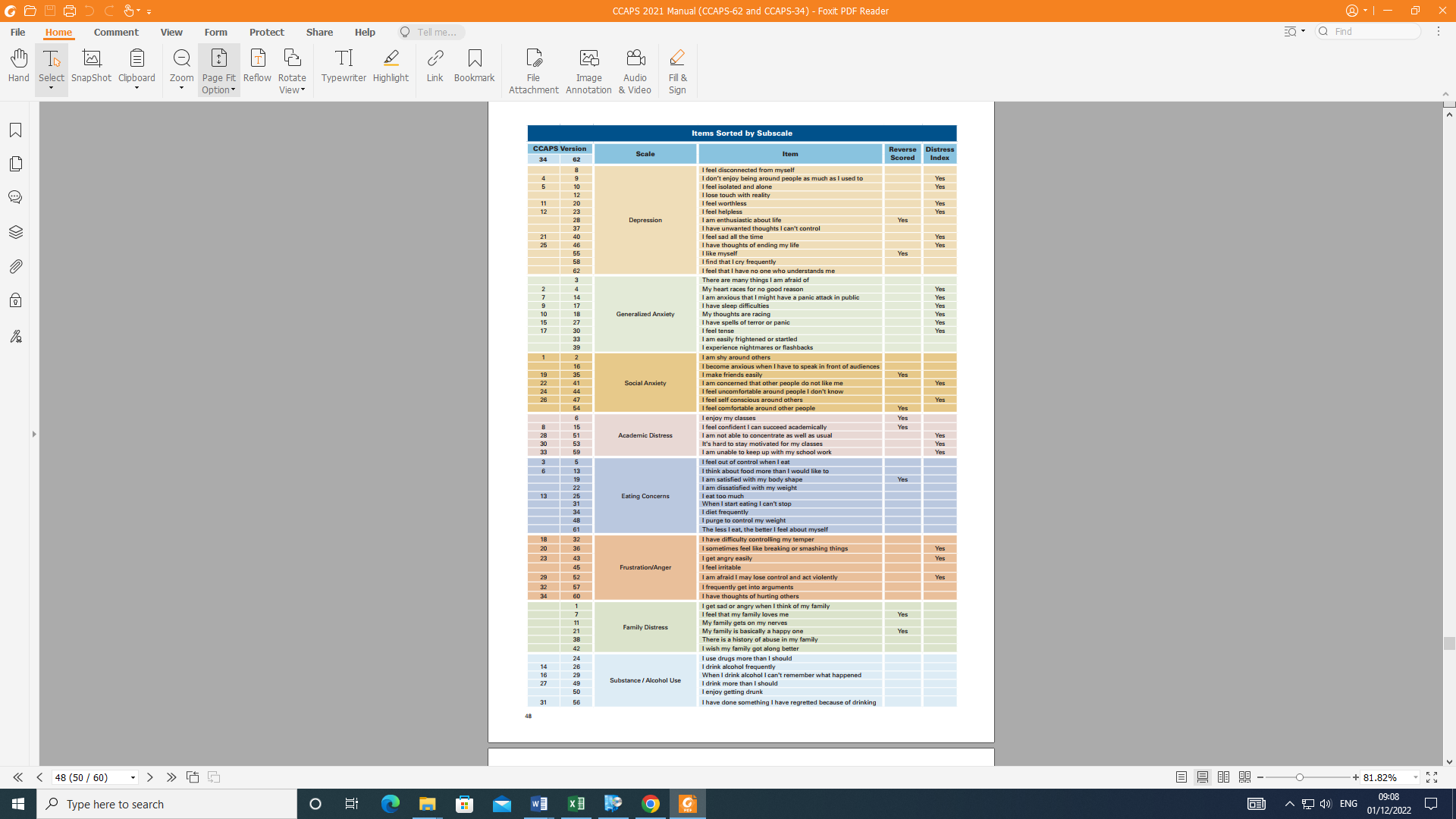

Supplement: Supplementary file 3 — Supplementary Material 3. [file 12913_2024_11204_MOESM3_ESM.docx]
