# Supplementary material for: Barriers to help-seeking, accessing and providing mental health support for medical students: a mixed methods study using the candidacy framework
Source: BMC Health Serv Res. 2024 Jun 15;24:738. doi: 10.1186/s12913-024-11204-8 (PMC11179297; doi:10.1186/s12913-024-11204-8)
Supplement: Supplementary file 4 — Supplementary Material 4. [file 12913_2024_11204_MOESM4_ESM.docx]

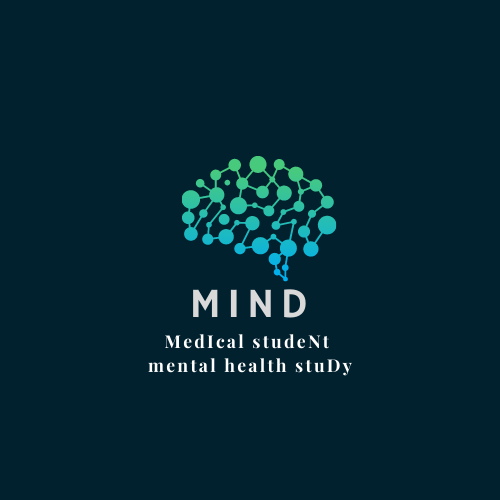


Additional File 4: Topic Guide for Professionals

MIND Topic Guide: Professionals

*Thank you for agreeing to take part in the interview today.*

*Check timing ok.*

*Check consent form filled in.*

*Are you happy for the interview to be recorded?*

*We’re interested in your views and experiences of providing mental health support to medical students studying at university.*

*We’d like to ask you what you know about providing mental health support – whether you’re a personal tutor, a family or friend, or a professional working at a student mental health charity, the university or an NHS service. We’d also like to ask you about your views on the challenges to providing this support to medical students and, if relevant, cross-partnership working with the university or NHS.*

*If running short on time, prioritise those with asterisk *.*

1. Can you tell me about [the service] and the kind of support you provide?
2. What is your role in [the service]?
3. In your role, what things are most important to know about providing mental health support?
4. *Do you know if medical students use your service?
   - [If yes] How often do you see medical students, on average?
   - [If yes[ Does [the service] provide mental health support to medical students throughout the academic year?
5. What’s your understanding of the experiences and academic journey of medical students?
6. *Who do you think is most likely to seek or access mental health support amongst medical students?
7. *What’s your understanding of the support that is available to medical students for their mental health? Does your service provide any support?
   - Is the support provided by [the service] appropriate for medical students’ mental health needs?
8. Is your role in managing a medical student’s mental health risk clear?
9. *How would you provide mental health support to medical students?
   - How confident do feel about discussing mental health with medical students?
   - Have you had any training in techniques to discuss mental health with medical students and if so, what?
10. How would you work with [the university / the Medical School or NHS] to support medical students?
    - Do you know how who to contact at the Medical School / university?
    - What are the important factors for providing effective mental health support for medical students?
11. *Is discussing mental health needs something you usually do with medical students?
    - When do you discuss their needs and why at that time?
12. Is discussing barriers/facilitators to help-seeking and accessing support something you usually do with medical students?
    - When do you discuss these barriers/facilitators and why at that time?
13. *What barriers or facilitators are you aware of when it comes to medical students seeking and accessing support, if any?
14. How do you record encounters with medical students?
15. What systems do you use for monitoring whether or not medical students have benefitted from support [the service] provided, if any?
16. How would you share this information with [the university / another healthcare provider]?
    - [If yes] Under what circumstances would you share this information?
    - [if yes] How often would you say this information needs to be shared?
17. *Do you see supporting medical student mental health as part of your role?
18. What do you consider a good assessment of medical students’ mental health?
19. Do you think support provided by [the service] is useful and appropriate for medical students?
    - [If not] Why not? Who or what service would be more appropriate?
    - [If yes] What makes it useful / appropriate for medical students?
20. *How difficult or easy is it for you to provide mental health support to medical students?
    - Does anything get in the way to providing this support or delivering the service?
    - What helps facilitate providing this support or delivering the service?
    - How does your relationship with [the medical school / university / NHS healthcare providers affect this?
21. How difficult or easy is it for you to work in partnership with [the medical school / university / NHS services]?
    - Does anything get in the way with working in partnerships across university and NHS support services?
    - What helps facilitate working in partnerships across university and NHS support services?
    - Are there any challenges with cross-partnership working?
22. How difficult or easy is it for you to talk about mental health in consultations with medical students?
23. *How difficult or easy is it to provide support for medical students who are not regularly on campus? (e.g. online learners, part-time students, international students)
24. *How confident are you that providing support as part of [the service] will improve mental health and wellbeing outcomes for medical students?

- How do you think [the service] impacts medical students’ mental health outcomes?

1. How confident are you that [the service] will improve medical students’ experiences of care?
2. What are the consequences of providing [the service] to medical students?
   - Does the service offer interventions, e.g. medication, therapies, follow ups, etc?
3. *How effective do you think the support provided by [the service] is in supporting a medical student’s mental health?
4. Why and when should a medical student use [the service?]
5. Are there any particular groups of medical students that you think [the service] is most appropriate for?
   - Who would you recommend [the service] to?
6. What is [the service] trying to achieve when providing support to medical students?
   - E.g. for a GP are you aiming to make an appropriate referral for IAPT, or are you hoping to improve mental health outcomes?
7. How do you hope [the service] develops for medical students?
   - Could [the service] be further developed or improved to meet medical students’ mental health needs? If so, how?
8. What are the incentives to improving mental health support provided to medical students? For example KPIs, service metrics
   - What might be achieved for [the service]?
9. Are there any aspects of medical student mental health that evoke an emotional response in you?
10. *To what extent do physical or resource factors facilitate or hinder your role in providing medical students with mental health support?
    - Can you tell me more about what resources you have used to provide this support, both individually and as a team?
    - Are there any resources your service is lacking when providing mental health support to medical students?
11. Do you have sufficient resources to work in partnership with [the medical school / university / other NHS healthcare providers] to support medical students’ mental health?
    - [If not] what resources do you need to facilitate partnership working?
    - Do you have the time and resources to develop this partnership?
12. To what extent do you feel supported in your role to manage medical students’ mental health?
    - Are there any people in your team who have been particularly influential?
    - Do you have any leadership in your area who is committed to student mental health?
13. How supported do you feel in managing medical students with high risk?
14. What training, if any, do you think should be provided to staff on how to support medical students and their mental health needs?
15. Do you have any training needs with the view of partnership working to enable effective management of medical student mental health and the risks?
    - [If yes] what are those training needs?

**THANK YOU**
