# Supplementary material for: Barriers to help-seeking, accessing and providing mental health support for medical students: a mixed methods study using the candidacy framework
Source: BMC Health Serv Res. 2024 Jun 15;24:738. doi: 10.1186/s12913-024-11204-8 (PMC11179297; doi:10.1186/s12913-024-11204-8)
Supplement: Supplementary file 5 — Supplementary Material 5. [file 12913_2024_11204_MOESM5_ESM.docx]

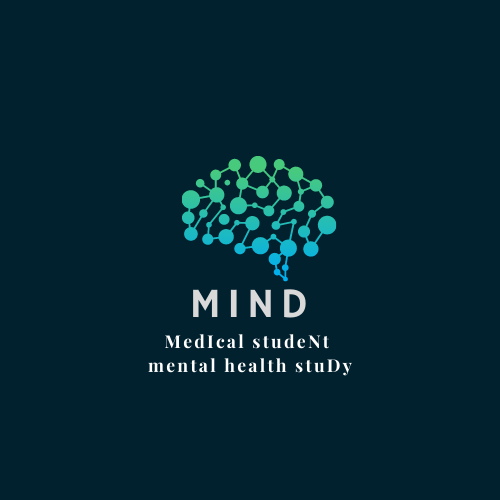

Additional File 5. Topic Guide for Medical Students

MIND Topic Guide: Students

*Thank you for agreeing to take part in the interview today.*

*Check timing ok.*

*Check consent form filled in.*

*Are you happy for the interview to be recorded?*

*We’re interested in your views and experiences of mental health support whilst studying medicine at university.*

*We’d like to ask you what you know about mental health services and support that is available, for example your personal tutor, friends and family, student mental health charities, university counselling services, NHS services like your GP, A&E, IAPT or any other mental health service you know about. We’d also like to ask you about your views on seeking help for your mental health whilst at university and, if you have accessed support, what your experience was like.*

*If running short on time, prioritise those with asterisk *.*

**Identification**

1. Can you tell me about your experience of studying medicine in Sheffield?
2. Can you tell me about the relationship between being a medical student and your mental health?
3. Have you experienced an episode of poor mental health before university?

- [If yes] Had you accessed support services for this episode before university?

1. Do you think your mental health is important?
2. At what point would you consider an episode of poor mental health, such as anxiety or stress, as being a problem? When would you seek help?
3. What would influence your decision to seek help if you’re experiencing an episode of poor mental health?
4. Do you think there is a stigma around mental health and using mental health services?

- [If yes] Is this stigma lesser or worse for medical students compared with other students not studying medicine?

**Navigation**

1. What mental health services are you aware of or know about in Sheffield?

- What university mental health services are you aware of?
- What NHS services are you aware of?

1. *Do you know what those mental health services are for, and the support they provide?
2. *How did you find out about those services?
3. *Would you know how to make contact with those services?

- How would you contact that service?

1. Whom would you contact first if you felt you were struggling with your mental health?

- E.g. Friends, family, university staff (academic or mental health support service) or NHS service

1. *Do you think mental health services are easily accessible to medical students?

- If not, why are they not easily accessible?

1. *Have you ever thought about accessing support, but then decided not to? If so, what were you reasons for not accessing support?
2. *Did anything get in the way of you seeking help for your mental health? For example your workload, timetable, low motivation etc…

- Fitness to practice, reputational damage, hearings and expulsion
- Representing failure

1. Did you have any concerns about fitness to practice when considering seeking help?
2. *What would help or enable you to seek support or access a mental health service?

- For example, deadline extensions, flexible placements, family/friends, personal tutor

**Permeability of services**

1. *Have you accessed any mental health services, university or NHS, whilst studying at university?

- This could be your GP, university mental health services, A&E

1. How easy did you find using this service?
2. How long did it take for you to be seen by someone? Did the service respond appropriately to your needs?
3. *Did anything get in your way when trying to access a service or receive support from that service after you made contact?

- For example workload, timetable, low motivation etc…

1. *Do you have the time to access support services?
2. Would you recommend these services to your peers?
3. Do you think that peers or support services would be understanding if you experienced an episode of poor mental health?
4. Have you ever delayed seeking or accessing support for your mental health?

- If so, why? And what point did you decide to access care? (e.g. crisis point?)

**Appearing at services and asserting candidacy**

[If a student has accessed a mental health service]

1. When did you access [x] service for mental health support?
2. *When you accessed this service, did anything get in the way of you receiving treatment or longer-term mental health support? For example, workload, placements, timetables

- Do you think there are barriers to receiving mental health support that are specific to medical students?

1. *When you accessed this service, did anything help or enable you to receive treatment or longer-term mental health support? For example, deadline extensions, flexible placements, family/friends, personal tutor
2. Were you told how to access support again in the future, if needed?
3. Did the healthcare professionals treat you with respect?

- Were you happy with the healthcare professional’s attitude and behaviour towards you?
- Did the healthcare professional appear competent?
- Were support services sensitive to any cultural or sexual identity issues?

1. Do you trust mental health support services?

- Do you trust your information will be kept confidential by the service?
- Do you have the same trust for NHS and university services?

1. How did you feel after accessing this service? Was it a positive or negative experience?

- If positive, why was it positive?
- If negative, why was it negative?

1. What could have been done to make your experience of the service better?
   - How could this service be improved for medical students who are accessing it?
2. Have you had any contact with occupational health? Did you find this helpful?

**Offers of, and resistance to, specific services**

1. Did you have any concerns about the support you were provided with?
   - Did you have any concerns about the medication you were prescribed, or any other support you were offered?
   - Were you satisfied with the treatment they offered, if any?
2. Was there any follow up or further treatment offered to you? (e.g. an appointment booked, or a follow up telephone call)
   - Do you think this follow up was appropriate, or met your mental health needs?
3. Did you **accept** the follow up or treatment that was offered to you?
   - [If refused] What were you reasons for not accepting the follow up or treatment offered?
   - Did you consider the follow up as unacceptable or unhelpful? Was your decision affected by a previous experience?
4. Did you **receive** the follow-up or treatment offered to you?
   - [For those in phase 2b onwards] Have placements affected your access to support? Did you receive continued support from [the service] during your placement?
5. Do you think that the service provided you with appropriate support at the right time?
   - Did you engage with the follow up or treatment? E.g. attend sessions, take medication, maintain contact with the service

**Operating conditions and local production of candidacy**

1. Do you think that you received the right mental health support at the right time?
   - Did this service meet your mental health needs as a medical student?
   - Do you think that the mental health service was suitable for you?
2. Would you use [x] service again if you were struggling with your mental health at university?
3. Is there anything you would change about the service that you used?
   - Is there anything that could be improved about the service?
4. Would you consider accessing a different mental health service in the future?
   - If so, which service would you access?
   - If not, why not?

**THANK YOU**
