# Supplementary material for: Barriers to help-seeking, accessing and providing mental health support for medical students: a mixed methods study using the candidacy framework
Source: BMC Health Serv Res. 2024 Jun 15;24:738. doi: 10.1186/s12913-024-11204-8 (PMC11179297; doi:10.1186/s12913-024-11204-8)
Supplement: Supplementary file 6 — Supplementary Material 6. [file 12913_2024_11204_MOESM6_ESM.docx]

# **Additional File 6: Statistical outputs**

**Analysis of CCAPS-34 scores and help seeking/service use questions**

The mean overall CCAPS-34 score of those who responded ‘yes’ to the question ‘Have you previously received mental health support before you started studying medicine at The University of Sheffield?’ is significantly higher than the mean of those who responded ‘no’ (unadjusted mean difference = 0.536, 95% CI= [0.291, 0.782]).

The mean overall CCAPS-34 score of those who responded ‘yes’ to the question ‘Have you previously received mental health support from The University of Sheffield counselling, NHS services* and/or a psychological wellbeing service whilst studying at University?’ is significantly higher than the mean of those who responded ‘no’ (unadjusted mean difference = 0.554, 95% CI= [0.310, 0.799]).

The mean overall CCAPS-34 score of those who responded ‘yes’ to the question ‘Are you currently receiving support from The University of Sheffield counselling, NHS services* and/or psychological wellbeing service?’ is significantly higher than the mean of those who responded ‘no’ (unadjusted mean difference = 0.624, 95% CI= [0.321, 0.927]).

The mean overall CCAPS-34 score of those who responded ‘yes’ to the question ‘Have you ever had concerns about your mental health and decided not to seek help from The University of Sheffield counselling, NHS services* and/or other psychological wellbeing services?’ is significantly higher than the mean of those who responded ‘no’ (unadjusted mean difference = 0.504, 95% CI= [0.279, 0.729]).

### **Analysis of CCAPS-34 subscale scores and help seeking/service use questions**

A one-way ANOVA was performed to compare the effect of the help seeking/service use questions on subscale scores. The questions were yes/no responses.

Table 1: Statistical output from the analysis of the additional question response against the subscale scores

| **Subscale score** | **Have you previously received mental health support before you started studying medicine at The University of Sheffield?** | **Have you previously received mental health support from The University of Sheffield counselling, NHS services and/or a psychological wellbeing service whilst studying at University?** | **Are you currently receiving support from The University of Sheffield counselling, NHS services and/or psychological wellbeing service?** | **Have you ever had concerns about your mental health and decided not to seek help from The University of Sheffield counselling, NHS services and/or other psychological wellbeing services?** |
| --- | --- | --- | --- | --- |
| **Academic distress** | F(1, 92) = [8.5248], p < 0.05 | F(1, 92) = [9.8196], p < 0.05 | F(1, 92) = [13.614], p < 0.05 | F(1, 92) = [10.085], p < 0.05 |
| **Alcohol** | F(1, 61) = [0.0465], p = 0.83 | F(1, 61) = [0.0309], p = 0.861 | F(1, 61) = [0.0001], p = 0.9932 | F(1, 61) = [0.1318], p = 0.7178 |
| **Depression** | F(1, 88) = [17.284], p < 0.05 | F(1, 88) = [19.918], p < 0.05 | F(1, 88) = [8.3184], p < 0.05 | F(1, 88) = [7.4861], p < 0.05 |
| **Eating concerns** | F(1, 51) = [0.0206], p = 0.8865 | F(1, 51) = [0.1585], p = 0.6922 | F(1, 51) = [0.4579], p = 0.5017 | F(1, 51) = [0.0495], p = 0.8248). |
| **Frustration** | F(1, 64) = [11.302], p < 0.05 | F(1, 64) = [0.4836], p = 0.4893 | F(1, 64) = [0.1302], p = 0.7194 | F(1, 64) = [0.0606], p = 0.8064 |
| **Generalised anxiety** | F(1, 94) = [19.673], p < 0.05 | F(1, 94) = [16.5], p < 0.05 | F(1, 94) = [17.524], p < 0.05 | F(1, 94) = [8.6966], p < 0.05 |
| **Social anxiety** | F(1, 94) = [0.4365], p = 0.5105 | F(1, 94) = [5.1427], p < 0.05 | F(1, 94) = [7.4159], p < 0.05 | F(1, 94) = [8.3221], p < 0.05 |

**Unadjusted mean difference (MD)**

Interpretation:

The mean overall score of those who responded ‘yes’ is significantly higher than the mean of those who responded ‘no’ (unadjusted mean difference = 0.637, 95% CI= [0.204, 1.071]).

### **Analysis of overall CCAPS-34 scores and subscales - demographics and help seeking/service use questions interaction effects**

Interactions between demographics and help seeking/service use questions were plotted against the overall and subscale scores, to visually inspect the data. This would highlight any obvious interactions to analyse. Some interactions between variables were suspected, however when the interaction models were fitted, no interaction terms were significant.
