# Supplementary material for: Barriers to help-seeking, accessing and providing mental health support for medical students: a mixed methods study using the candidacy framework
Source: BMC Health Serv Res. 2024 Jun 15;24:738. doi: 10.1186/s12913-024-11204-8 (PMC11179297; doi:10.1186/s12913-024-11204-8)
Supplement: Supplementary file 7 — Supplementary Material 7. [file 12913_2024_11204_MOESM7_ESM.docx]

### **Additional File 7: Floor and ceiling effects**

Floor and ceiling effects were descriptively summarised for each subscale. Floor effects refer to participants who scored 0 for all questions in that subscale, whilst ceiling effects refer to participants who scored 4 for all questions in that subscale.

Table 1: Floor and ceiling effects for each subscale

|  | **Floor effects** | **Ceiling effects** |
| --- | --- | --- |
| **Academic distress** | 3 (2.94%) | 2 (1.96%) |
| **Alcohol** | 37 (36.27%) | 0 (0.00%) |
| **Depression** | 12 (11.76%) | 0 (0.00%) |
| **Eating concerns** | 33 (32.35%) | 4 (3.92%) |
| **Frustration** | 35 (34.31%) | 0 (0.00%) |
| **Generalised anxiety** | 6 (5.88%) | 0 (0.00%) |
| **Social anxiety** | 0 (0.00%) | 1 (0.98%) |

The denominator is the total number of participants with complete questionnaire data (102 participants, 1 participant had no questionnaire data).
